# A Review of Intelligent Assembly Technology of Small Electronic Equipment

**DOI:** 10.3390/mi14061126

**Published:** 2023-05-26

**Authors:** Wei Tian, Yifan Ding, Xiaodong Du, Ke Li, Zihang Wang, Changrui Wang, Chao Deng, Wenhe Liao

**Affiliations:** 1 College of Mechanical and Electrical Engineering, Nanjing University of Aeronautics and Astronautics, Nanjing 210016, China; 2 No. 29 Research Institute of CETC, Chengdu 610036, China; 3 School of Mechanical Engineering, Nanjing University of Science and Technology, Nanjing 210094, China

**Keywords:** small electronic equipment, intelligent assembly technology, visual localization, path and trajectory planning, force–position coordination control

## Abstract

Electronic equipment, including phased array radars, satellites, high-performance computers, etc., has been widely used in military and civilian fields. Its importance and significance are self-evident. Electronic equipment has many small components, various functions, and complex structures, making assembly an essential step in the manufacturing process of electronic equipment. In recent years, the traditional assembly methods have had difficulty meeting the increasingly complex assembly needs of military and civilian electronic equipment. With the rapid development of Industry 4.0, emerging intelligent assembly technology is replacing the original “semi-automatic” assembly technology. Aiming at the assembly requirements of small electronic equipment, we first evaluate the existing problems and technical difficulties. Then, we analyze the intelligent assembly technology of electronic equipment from three aspects: visual positioning, path and trajectory planning, and force–position coordination control technology. Further, we describe and summarize the research status and the application of the technology and discuss possible future research directions in the intelligent assembly technology of small electronic equipment.

## 1. Introduction

With the rapid development of electronic technology, electronic equipment has penetrated all aspects of people’s lives, as well as national defense. Electronic equipment plays an important role, from mobile phones and computers to satellites and radars [1]. In addition, as the primary battle weapon of information confrontation and the soul of modern weapons, the development level of electronic equipment is an essential reflection of a country’s military strength and the development focus of national defense strategy [2]. Electronic equipment, represented by communication and navigation equipment, radar, supercomputer, high-end network equipment, etc., is characterized by a complex structure, short development cycle, fast replacement, multiple varieties, and high-reliability requirements [3]. With the rise of a new technological revolution and industrial reform, electronic equipment is also developing in the direction of functional integration, miniaturization, and compact structure.

Assembly is the final stage and a critical technical step of electronic equipment manufacturing; its quality is directly related to the production efficiency, performance, and cost of electronic equipment. Electronic equipment parts have various types, complex shapes, different assembly requirements, and high precision requirements [4]. However, parts are still assembled manually and there are problems such as poor assembly positioning accuracy, low production efficiency, poor consistency of batch production, and proneness to accidents. A small amount of tooling equipment can replace workers to complete simple and tedious operations and improve assembly efficiency. However, there are still shortcomings, such as high cost, insufficient capacity for large-scale assembly, etc., which makes it challenging to respond rapidly to changes in the type of assembly parts and process flow. At the same time, electronic equipment manufacturing is a typical multi-variety small-batch mode, which requires outstanding flexibility in the assembly process. Therefore, from the perspective of the future development direction of electronic equipment assembly, traditional assembly methods face challenges in meeting the needs of electronic equipment production because of the high complexity and high precision requirements of electronic equipment assembly. With the rapid development of Industrial 4.0 and Assembly 4.0, the intelligent assembly technology of electronic equipment has become a new development direction of electronic equipment assembly technology.

Industry 4.0 was initially proposed in Germany at the Hannover Messe in 2013. One of its goals was to develop innovative high-quality products with a shorter time-to-market through intelligent production and assembly (Assembly 4.0). Then, China, the United States, Japan, etc., all proposed similar concepts. Assembly systems have also moved into the era of Industry 4.0 and developed into Assembly 4.0 [5,6]. Berglund and Daneshmand pointed out that Assembly 4.0 mainly includes algorithm optimization, augmented reality-assisted assembly, robot-assisted assembly, path planning, compliance control, etc. [7,8]. Intelligent assembly, that is, Assembly 4.0, can not only effectively solve the problems of small batches and high flexibility requirements in the assembly of electronic equipment components but can also improve assembly accuracy, shorten development cycles, and reduce research and development costs, supporting the transformation of electronic equipment from small-scale automated manufacturing to large-scale intelligent assembly. Therefore, intelligent assembly has become a meaningful way to improve the automation and intelligence level of electronic equipment parts’ assembly. It is also a difficult hotspot in the current research field of robot assembly and intelligent manufacturing, which has attracted extensive attention from experts and scholars. To date, there have been comprehensive reviews about intelligent assembly. For example, Dong et al. [9] reviewed the intelligent assembly of industrial robots in the field of aerospace. Zhao et al. [10] summarized and analyzed the docking assembly of large aircraft segments. Xu et al. [11] summarized the latest research on robot-guided assembly technology. Jiang et al. [12] investigated intelligent robot systems for large-scale spatial structures’ on-orbit assembly and outlined modular assembly and on-orbit manufacturing technologies.

**Figure 1:**
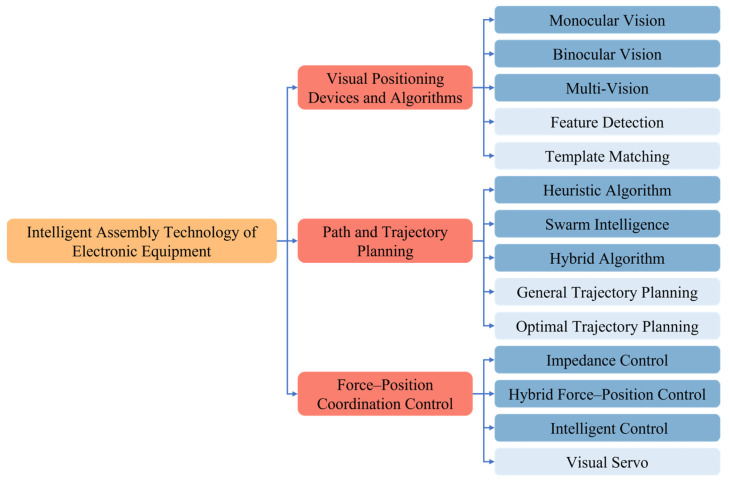
Key technologies of intelligent assembly of electronic equipment.

The above reviews provided references for the research of automated intelligent assembly and made specific contributions to their respective research fields. However, there are no summaries of research on the intelligent assembly of electronic equipment. Therefore, starting from the requirements in the area of intelligent assembly of electronic equipment, this article systematically summarizes the research and applications related to positioning equipment and algorithms (Section 2), path and trajectory planning (Section 3), and force–position coordination control (Section 4) that are involved in the intelligent assembly of electronic equipment, as shown in Figure 1. Finally, this paper offers prospects for the future development of intelligent assembly of electronic equipment (Section 5).

## 2. Positioning Equipment and Algorithms

**Figure 2:**
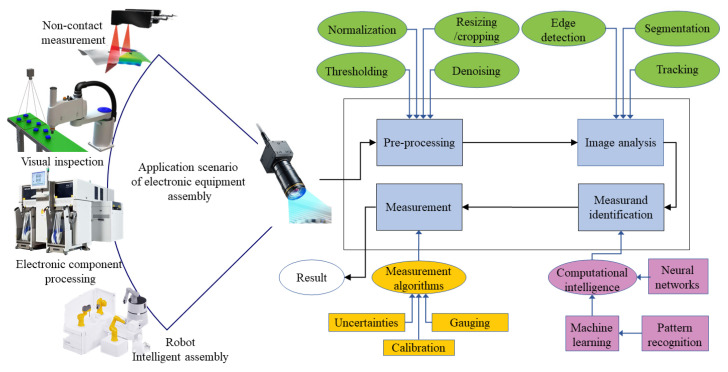
Application flow of machine vision [15].

With the development of the continuous demand for production automation and intelligence, object recognition and positioning based on machine vision has become one of the current research hotspots. Machine vision has a wide range of application scenarios, such as the drilling and riveting of large aerospace panels [13], automatic assembly of array antenna, intelligent mounting, and welding of component circuits [14], etc. Many fields of assembly processing involve the recognition and positioning of target objects. In the intelligent assembly process of electronic equipment, machine vision performs the information collection of micro/nano components. Then, the information is processed by the computer to complete the identification and positioning of the target object, guide the actuator to make relevant actions on the target object, and finally realize the automatic and intelligent production process. The usage scenarios and application processes of machine vision are shown in Figure 2 [15]. In the above processes, positioning equipment and algorithms are the basis for ensuring high-quality assembly. Compared with the mechanical positioning system of traditional machine and pipeline processing equipment, more efficient machine vision positioning technology significantly improves electronic equipment’s assembly efficiency and accuracy. Optical measurement technology has many advantages, such as strong real-time performance, high precision, and a high degree of automation. This section mainly focuses on visual positioning technology and describes the positioning equipment and algorithms of the intelligent assembly of electronic equipment.

### 2.1. Positioning Equipment

The visual positioning system is widely used in intelligent and automatic assembly equipment because of its non-contact measurement method and strong real-time performance. Traditional machining obtains positioning coordinates through the probe, positioning block, and other equipment or uses the processing origin of the two-dimensional drawings of the workpiece for positioning. This method often has high requirements on the processing quality of the workpiece and the machine fixtures and the accuracy of the positioning data is inferior. It only suits workpieces with low processing requirements and high batch consistency. Due to electronic equipment’s unique function, its internal components’ geometric features are also exceptional and the batch consistency of this kind of special workpiece is poor. In addition, the size of components in electronic equipment is mainly on the micro/nano scale and traditional positioning methods are not suitable for positioning. On the contrary, a visual positioning system can obtain the object’s size, position, attitude, and other geometric parameters to be processed in the assembly process through video image information and feature extraction algorithms, which can better meet the positioning requirements in the assembly process. Visual positioning equipment is classified according to the number of cameras, which can be divided into monocular vision, binocular vision, and multi-vision.

#### 2.1.1. Monocular Vision Camera

**Figure 3:**
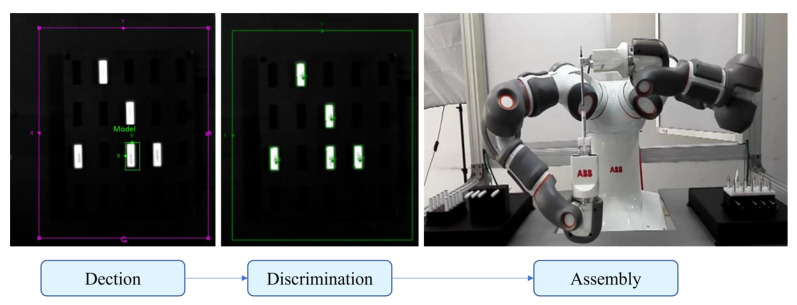
Part recognition and assembly based on monocular vision [23].

Monocular vision cameras are widely used in robot obstacle avoidance, industrial detection and positioning, and target tracking because of their advantages, such as simple equipment, high integration, small size, and low cost. Li [16] solved the problem of visual recognition and positioning of small-sized threaded holes in the process of automatic bolt assembly through monocular vision. Yu et al. [17] combined monocular vision and laser displacement sensors to solve the problem of difficult identification and positioning of scattered rivets in the process of automatic nail feeding. The recognition accuracy rate was above 85% and could adapt to 30~70% of the lighting brightness changes, significantly improving the drilling and riveting efficiency. Bui et al. [18,19] used the parallax method to calculate two images at two different optical axis positions. They measured any object using the parallax generated between the images. Even if the geometric size of the shooting target was unknown, it could be accurately measured. Guan et al. [20] proposed a monocular vision positioning method for tiny workpieces. Using a monocular camera, the displacement and rotation angle of workpieces of different shapes were measured with high precision. Liu et al. [21] used a monocular vision camera to locate the shaft and the hole. Using image features, they successfully calculated the tilt angle between the shaft and the hole. Wang et al. [22] used a monocular camera to detect straight-lined and curved features. Combined with the workpiece database, they achieved an excellent positioning effect and provided adequate grasping information for industrial robots. In order to solve the assembly problem of small parts in electronic products, Fang et al. [23] developed a system using a dual-arm robot for assembly (Figure 3). The system used a monocular camera to identify the location of the small parts. The experimental results demonstrated the effectiveness of the system.

Compared with binocular vision [24], multi-vision [25,26], and depth-vision systems [27], the monocular-vision system [28,29] has the advantages of simple calibration, low cost, and high flexibility. Therefore, monocular vision is mainly used in intelligent assembly for pose estimation, target identification without depth information, and hand–eye calibration, for example, calibration of the stereo target [30], measurement of the 3D pose of the workpiece [31], obstacle avoidance detection in the process of processing and assembly [32], etc.

#### 2.1.2. Binocular Vision Camera

**Figure 4:**
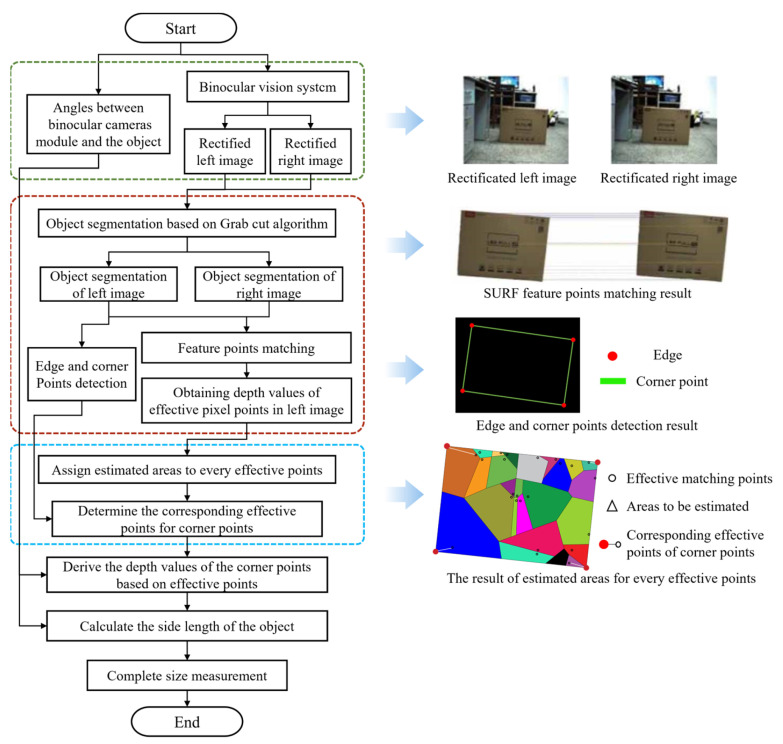
Object size measurement based on the MBC (“M” refers to “monocular vision”, “B” refers to “binocular vision”, and “C” refers to “cooperation”) method [35].

Binocular measurement technology simulates the measurement method of human eyes. It is the most widely used measurement method to calculate the three-dimensional position coordinates of the measured object by photographing the two-dimensional plane information of different angles of the same thing. In the production process, objects have different appearances, shapes, attitudes, illumination and occlusion, and other complex conditions, so it is necessary to realize the rapid detection and positioning of objects in production. Li et al. [33] used binocular vision for position estimation, effectively enabling the robot to grasp objects. Ma et al. [34] studied an image-guided circular hole positioning method based on industrial robots and realized the high-precision detection of circular hole workpieces’ parameters and poses. Experiments showed that the accuracy of the distance between the hole position and the hole center was maintained within 1 mm and the accuracy of the pose of the hole was maintained within 0.3°. Zhou et al. [35] proposed a camera internal collaborative measurement system based on the cooperation of monocular and binocular vision. Experimental results showed that the absolute relative measurement errors of this method were lower than 3.5%. The flow chart is shown in Figure 4. Kalogeiton et al. [36] used stereovision to navigate, locate, and define the surrounding environment and avoid any possible obstacles, following the best route in the map area. Experiments proved that this method enabled mobile robots to quickly complete tasks. Lu et al. [37] extracted the features of riveting holes based on the region growing method and used the binocular vision camera to locate and calculate the pose of the hole. Experiments verified that this method could accurately identify the posture of a riveting hole with a diameter of 4 mm.

Binocular vision measurement has the advantages of appropriate accuracy and simple system structure, suitable for non-contact target detection and positioning. In the field of intelligent assembly of electronic equipment, it is widely used in three-dimensional reconstruction [38], target positioning [39], and trajectory planning [40].

#### 2.1.3. Multi-Vision Camera

**Figure 5:**
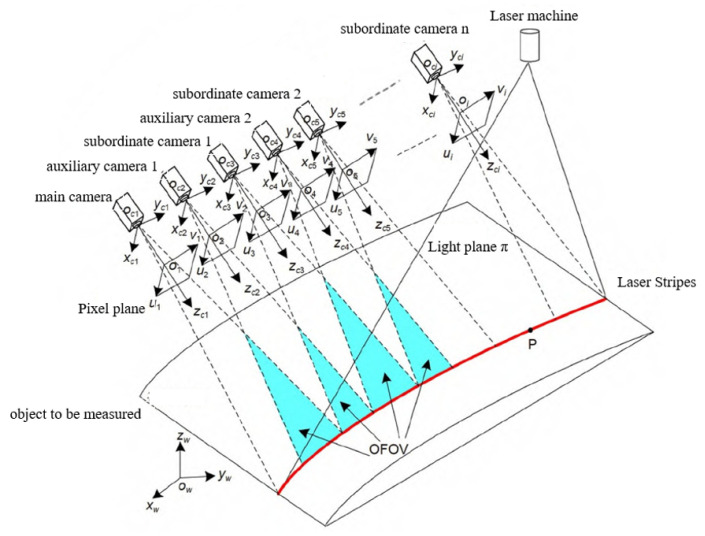
Global calibration of the multi-vision line structured light measurement system based on auxiliary cameras [44].

Currently, most of the vision systems used in industrial production are based on monocular or binocular structures, which can complete simple visual tasks. However, with the improvement of production standards in modern industry, especially in industrial production lines, traditional monocular and binocular vision systems cannot meet the requirements of accurate large-scale detection; however, multi-vision vision systems can achieve such requirements. Ma et al. [41] proposed an ellipse detection algorithm based on gradient and position by designing the ring code mark points and positioned the mark points through multi-eye vision. After experimental analysis, the error of this positioning method was below 0.05 pixels and could be used for multi-target positioning in the assembly process. Xu et al. [42] combined 3-HSS (H: helical/screw pair, S: spherical pair) parallel robots to design a multi-eye camera vision system, including two binocular cameras and one monocular camera, which could complete the tasks of real-time positioning and capturing in industrial production. Xia et al. [43] proposed a global calibration method suitable for multiple cameras. Experiments showed that it had the advantages of high accuracy (root mean square error of 0.04 mm) and low cost, which was especially suitable for on-site calibration in industrial production. Aiming at the difficulty of calibrating multi-view visual cameras, Ge et al. [44] proposed a global calibration method of a multi-view visual line structured light measurement system based on auxiliary cameras that did not require the help of high-precision measuring equipment and complex targets, as shown in Figure 5. Experiments showed that the global calibration error of this method was 0.067 mm, which effectively improved the measurement accuracy and provided an accuracy guarantee for the subsequent large-component robot “measurement-machining” integrated operation.

Multi-vision is developed based on monocular and binocular vision technologies and there are already cases of its application in assembly [45,46]. Multi-vision can achieve higher precision spatial positioning, but the calibration requirements are more complicated than monocular or binocular vision. We summarize the advantages and disadvantages of the three positioning equipment, as shown in Table 1.

### 2.2. Positioning Algorithms

In the intelligent assembly of electronic equipment, positioning algorithms are equally important, such as in the surface circuit processing of conformal antenna, the insertion of array antenna, the cooling structure assembly of a small chip, etc. It is worth noting that, whether it is the processing of the conformal antenna’s surface circuit or the array antenna’s insertion, the size of these components and structures is often very small. Taking PCB (printed circuit board) components as an example, the size of the 0201 resistor commonly used in antennas is within 0.12 mm^2^, which puts quite high requirements on the positioning algorithm.

**Figure 6:**
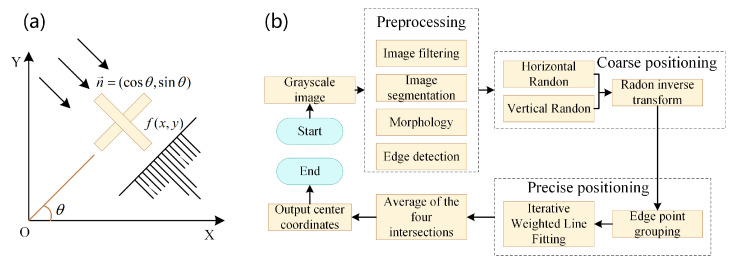
PCB cross-positioning feature recognition based on the improved feature detection algorithm: (**a**) the principle of Radon transformation; (**b**) flow chart of Radon cross-positioning feature recognition algorithm [53].

Generally, feature detection algorithms are used for calibration and positioning. The feature detection algorithm obtains target parameter information through mathematical operation using the target’s shape characteristics, size parameters, and other information. It has a good application scenario in AOI (automatic optical inspection) detection, PCB positioning, and positioning of the patch components of the SMT (surface mounting technology) machine. Xi et al. [47] proposed to obtain the pin centroid of IC (integrated circuit) components based on the region growth method and used the least square fitting line to fit the pin centroid of the component to obtain the component’s offset correction displacement and angle, achieving high-precision processing of the placement machine. Gioi [48] proposed a linear-time line segment detector (LSD), which was faster and less complex than Hough transform. Yu et al. [49] proposed a perceptually accurate line segment detection approach (PLSD), which used line segment grouping, line segment verification, and a multi-scale framework, to improve the line segment detection method and generate higher quality line segments. White et al. [50] obtained welds based on least squares and gravity center fitting and extracted welding points to realize weld positioning. Yang et al. [51] used the least square method to extract the linear features in the environment. The robot’s self-positioning accuracy was improved based on the linear features and combined with the ICP (iterative closest point) algorithm. Jiang et al. [52] used the improved least square method to conduct linear fitting on the edge of the chip element. They obtained the position error of the chip element through matrix fitting, which met the requirements of high precision and high speed of the positioning detection system. The improved Radon positioning algorithm proposed by Zhou [53] and the LSD-based positioning algorithm had vital accuracy, stability, and anti-interference, which realized more accurate positioning of PCB cross reference points, as shown in Figure 6. Chen et al. [54] combined phase consistency and Hough circle detection to realize the high-precision location of circular mark points on the PCB board. This method had strong anti-interference ability and a low requirement on image brightness. He et al. [55] improved the RANSAC (random sample consensus) algorithm through constrained random selection points and least square optimization fitting, which improved the processing accuracy and efficiency of the solder paste printing machine. Ayala-Ramirez et al. [56] proposed to detect circles based on the genetic algorithm (GA). This method could detect circles with sub-pixel accuracy and had good detection results for obscured or imperfect circles. Mantau et al. [57] proposed an optimized particle swarm optimization algorithm (PSO) for image ellipse detection. Zhang et al. [58] proposed an improved Hough transform method based on the simulated annealing algorithm, which improved the calculation speed of the Hough transform and quickly and accurately obtained the diameter of the red-hot circular workpiece.

**Figure 7:**
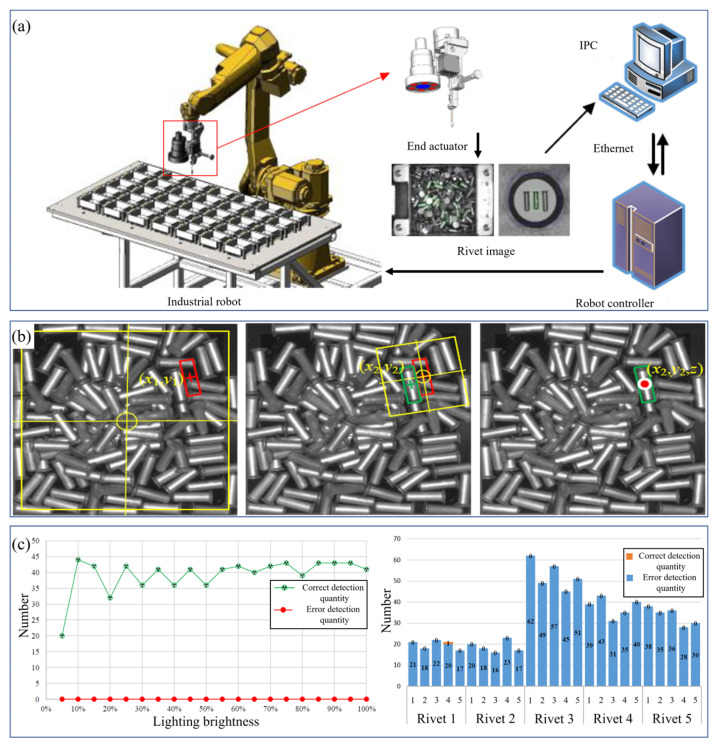
Micro rivet identification and positioning based on template matching: (**a**) automatic nail feeding system; (**b**) monocular visual inspection of automatic nail feeding system; (**c**) micro rivet recognition results based on the template matching algorithm [62].

In electronic equipment, many small-size machining parts need pose adjustment or spatial positioning. These parts are often not apparent in shape and, because of their small size, it is not convenient to use cooperative targets for identification and positioning. Conventional feature detection cannot meet the processing requirements, so template matching has become a standard solution. Korman et al. [59] extended template matching to deal with arbitrary two-dimensional affine transformations by discretizing the affine space and hierarchical search strategy. Crispin et al. [60] proposed a general template method based on GA to solve the inspection problem of multiple components on PCB. A generalized grayscale modal template image was generated by extracting a set of template images of the same size for each component to be located and identified. The average of the corresponding pixel values in each template image was calculated. However, this approach presented the problem that all components must be placed in the same direction. This meant that all components were placed horizontally or vertically; otherwise, generalized templates were hardly helpful in handling maximum likelihood searches. Multiple template matching was required when various components were placed horizontally and vertically rather than a single generalized template. To solve this problem, Wu et al. [61] proposed a normalized cross-correlation based multi-template matching (MTM) method to solve the problem of multi-component PCB detection. The MTM method only focused on the most likely template and component placement direction did not affect identification and positioning. Experimental results showed that the proposed algorithm successfully located all the components on the circuit board in all operations and the calculation was more minor and had higher efficiency. Zhuang et al. [62] proposed a micro rivet positioning method based on heterogeneous sensor data fusion to identify and position scattered rivets in an automatic nailing system. The method realized accurate measurements of the critical size of the micro rivet through template matching and improved the efficiency and reliability of the nailing system. The composition of the system is shown in Figure 7a and the recognition effect is shown in Figure 7b,c.

Zhang et al. [63] proposed a template matching method (weighted smallest deformation similarity (WSDS)) to solve problems if the camera lens was loose or dirty, the brightness of the light source changed, or part of the image transmission was lost during the SMT process, which could be applied to the rough positioning of components in some exceptional cases. Liu et al. [64] realized multi-template matching by introducing a PPL parallel library and improved the search efficiency of the template-matching pyramid search algorithm by 56.3%. Xie et al. [65] realized rapid matching and accurate positioning of components in patching by template matching to solve the detection work of components lacking markers in the patch process. Xiong [66] proposed a template-matching algorithm based on edge information for special-shaped patches and used a pyramid search strategy instead of a traditional search strategy to speed up the algorithm’s running time. The algorithm had the advantages of universality, fast running, and high precision for special-shaped patches. Yan et al. [67] improved the accuracy and real-time performance of target region detection and positioning for high-speed printed circuit board assembly by conducting a four-layer Gaussian pyramid transformation to match image and template images and to optimize search areas with the particle swarm optimization algorithm.

As the assembly of electronic equipment changes from manual to intelligent and automatic assembly, machine vision is increasingly applied in intelligent assembly production lines because of its advantages. Different electronic devices have different dimensions, processing requirements, and processing processes, so the positioning method and algorithm in the assembly process must also be selected according to the specific equipment. In the future, the electronic assembly will develop towards the direction of easy perception of the state, high machining accuracy, and high assembly efficiency, so the research and improvement of positioning equipment and positioning algorithms must also develop towards achieving accuracy and efficiency.

## 3. Path and Trajectory Planning

Modern electronic equipment is widely used in communication, navigation, detection, and other aspects. No matter what kind of equipment it is, many PCBs are used to carry various functions and sizes of electronic components. They also bear the signal transmission, power supply, and other functions. Therefore, as the essential component of electronic equipment, the processing and assembly of PCBs becomes a crucial part that directly affects the performance of electronic equipment. This section will focus on the research status of PCB processing and detection path planning and analyze the trajectory planning technology of industrial robots used in the PCB assembly process.

### 3.1. Machining Path Planning

The primary and core components of electronic equipment, PCB assembly, and processing are receiving more and more attention from researchers. The production process of PCBs is quite complex, including cutting, exposure, etching, lamination, drilling, mounting, welding, AOI detection, etc. Among them, the time spent on drilling occupies 70% of the whole PCB processing time [68]; the speed of mounting and AOI detection also determines the time efficiency of PCB processing to a large extent [69]. Therefore, the key to improving the overall PCB processing efficiency lies in the time optimization of the hole processing path, mounting sequence, and AOI detection path. Many scholars have carried out research in this field. The time optimization problems mentioned above can be combined into the traveling salesman problem (TSP). Take the symmetric TSP problem as an example; this problem assumes that a salesman needs to go to n cities to sell products and searches for the shortest path that starts from one of the cities, passes through all the cities only once, and returns to the starting point. The distance between two cities, i and j, is denoted as $d_{i,j}$. The symmetric TSP problem assumes that for any i,j, $d_{i,j}=d_{j,i}$, otherwise it is called an asymmetric TSP problem [70]. The above problems can be converted into the following optimization problem:

$$Dist_{min}=\overset{n}{\underset{i=1}{\sum }}\overset{n}{\underset{j=1,i\neq j}{\sum }}k_{i,j}d_{i,j}$$

where $k_{i,j}$ indicates whether this path connects cities i and j and when city i is connected to city j, $k_{i,j}\;$= 1, otherwise $k_{i,j}$ = 0.

The TSP problem is a typical NP (nondeterministic polynomial) problem. A non-deterministic algorithm can solve it in polynomial time, but it has not been proven that all NP problems have polynomial-time deterministic algorithms [71]. This problem can test whether the answer is correct in a finite time, but it cannot guarantee that the correct solution can be found in a limited time. There are two main types of algorithms to solve this kind of problem: deterministic algorithms and intelligent algorithms. Deterministic algorithms include the dynamic programming method [71,72], the branch and bound method [71,73], the cutting planes method [74], etc. These algorithms can find exact solutions, but the time and space complexity will increase sharply with the increase of the problem’s scale, so they are only suitable for solving small-scale problems. Intelligent algorithms include the genetic algorithm [75], the ant colony algorithm [76], the particle swarm optimization algorithm [77], the hybrid algorithm [78,79], etc. Although these algorithms cannot guarantee exact solutions, they can quickly find approximate solutions within the allowable error range, achieving the best balance between solving accuracy and solving efficiency. Intelligent algorithms are often used to obtain approximate solutions when solving the time optimization problems of drilling, mounting, and AOI detection of PCBs.

Although the problems above can be classified as TSP problems, there are many differences in solving them: drilling path optimization is a two-dimensional symmetric TSP problem that requires a fixed tool change point as the starting point; mounting path optimization is a three-dimensional asymmetric TSP problem [80]. AOI detection needs to consider the clustering problem of image acquisition windows and the influence of the change of center position of image acquisition windows on path length. This section elaborates on PCB hole processing path planning, mounting sequence optimization, and AOI detection path planning. We clarify the differences among the three TSP problems and the development process of corresponding solutions. We also summarize the relevant technologies at the end.

#### 3.1.1. Drilling Path Optimization of PCBs

**Figure 8:**
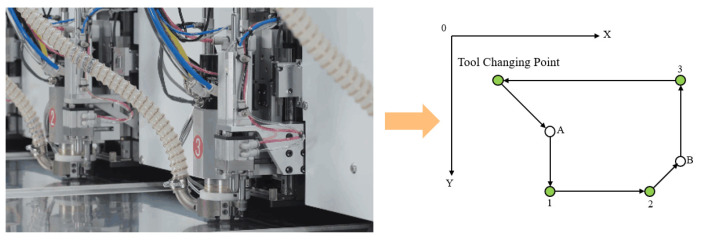
Example of PCB NC drilling machine and hole processing path.

Drilling is an essential process in PCB production that plays the role of electrical connection and fixing devices; generally, mechanical drilling or laser drilling is used. Due to the high cost of laser drilling and the limitation of processing materials, a PCB NC drilling and milling machine is often used to drill holes and laser drilling is used when processing many micro holes. When mechanical drilling is used, tool changing is required at the tool changing point according to the aperture to be drilled, so hole machining path planning is equivalent to a symmetric TSP problem with a fixed starting point. When drilling PCB, the XY axis of the drilling machine generally runs simultaneously at the highest speed to pursue the highest processing efficiency. The maximum speed of the two axes is the same. Therefore, the drilling machine usually does not run in a straight line between two points [81]. As shown in Figure 8, when running from tool change point 0 to drilling point 1, the X axis first reaches point A. Then, the X axis stops moving and the Y axis moves to point 1. Therefore, whether the path should be evaluated using empty travel distance or time is also worth considering.

**Figure 9:**
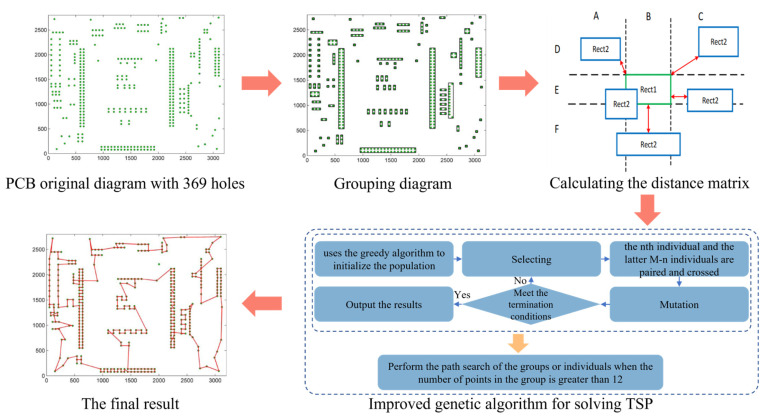
An improved genetic algorithm for PCB drilling path optimization based on a full combination breeding strategy (FCGA) [88].

The time of PCB hole processing consists of two parts: $T_{Drill}$ and $T_{Travel}$. $T_{Drill}$ increases linearly with the number of holes; its running time is related to hole type and drilling machine, so it is not the optimization objective to be concerned in this paper. $T_{Travel}$ often takes up more than half of the entire processing time; this needs to be optimized [82]. Therefore, reasonable path planning is essential. In 1983, Metelco (a Greek PCB manufacturer) urgently needed to solve the problem of highly time-consuming PCB drilling to improve its competitiveness in the European high-tech market. Designing an efficiently coded compound heuristic algorithm instead of the determination algorithm increased PCB production by 10% and saved the company over $10,000 per year in employees’ expenses [83]. At the same time, Northern Telecom Electronics Inc. also conducted a similar study using the nearest neighbor heuristic algorithm and clustering algorithm to shorten the path length and travel time [84]. However, the above algorithm used the Euclidean distance to measure the solution, which differed from the drilling machine’s actual route, so the path length estimation would be biased. To solve the above problems, Wang et al. [81] used the minimum empty travel time as the objective function to replace the minimum empty stroke time. They used the simulated annealing algorithm to solve the minimum empty travel distance, further improving the processing efficiency. Wang et al. [84] improved the greedy algorithm. They combined it with the ant colony algorithm to solve the problem of many empty strokes caused by the direct greedy algorithm and increased the processing efficiency by 23.9%. Wei et al. [85] used the improved genetic algorithm to solve the path optimization problem of PCB hole machining. By introducing the greedy crossover operator, inversion mutation, and exchange mutation operator, they obtained satisfactory optimization results and shortened the drilling path by 19.08%. However, the global convergence of the earlier intelligent algorithm was not good and it was easy to fall into the local optimal solution. Zhu [86] established a globally convergent particle swarm optimization algorithm, regenerated the particles that had stopped converging to improve the global convergence ability of the algorithm, and introduced the order exchange unit (OEU) and the order exchange list (OEL); this made the new algorithm easy to implement and fast in convergence. Adam et al. [87] used the global particle swarm optimization algorithm. They introduced the dynamic linear decreasing inertia weight to obtain calculation results similar to those in the literature [86], while spending less computing resources. The above methods optimized the algorithm’s convergence to a certain extent but were only suitable for solving small-scale drilling path optimization problems. As the number of holes increased, they faced issues of poor optimization results and reduced efficiency. Wang [88] proposed a grouping strategy algorithm based on an adjacency list, which decomposed large TSP problems into several small TSP problems, and used the improved genetic algorithm based on the full combination breeding strategy (FCGA) to solve the small TSP problems, respectively. Experiments showed that the algorithm significantly reduced the number of iterations and the drilling running time was shortened by 17.04% on average. The large TSP problem was well solved. The specific principle of this algorithm is shown in Figure 9. Zhang et al. [89] adopted the k-means algorithm and the improved simulated annealing algorithm to improve convergence and shortened the shortest path by 17.39%, aiming at the problems of a large number of micro holes and uneven distribution density on the new-generation PCB board (the HDI (high density interconnector) board). Huang [90], aiming at the limitations of existing SOM (self-organizing map) algorithms in solving large-scale problems, adopted the solution idea of multi-level reduction and step-by-step merging for large-scale problems and proposed an ASOM-MR hybrid algorithm to effectively solve large-scale PCB drilling problems.

#### 3.1.2. PCB Mounting Sequence Optimization

SMT is a technology that places components onto a PCB board that has been coated with solder paste and connects components to the PCB board through soldering. The SMT production line is the essential equipment to realize the above process, including feeding machine, printing machine, SMP (surface mounting placement) machine, reflow welding machine, etc. The core equipment is the SMP machine and the mounting speed is directly related to the production efficiency of a whole SMT production line [91]. The key factors affecting the mounting efficiency of the SMP machine are the component mounting sequence, the component position distribution on the feeder, and the motion control. Among them, the first factor can be regarded as a three-dimensional asymmetric TSP problem and the second factor as a quadratic distribution problem [92], both of which belong to the above NP problems and are difficult to solve.

**Figure 10:**
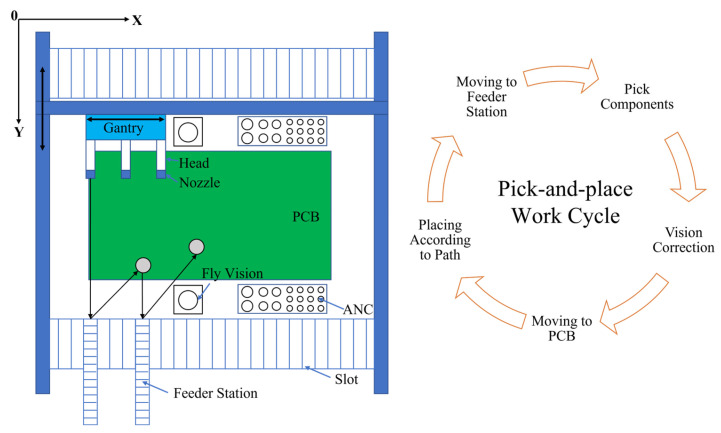
Schematic diagram and working cycle of the arch-type mounting machine.

The types of SMP machines can be roughly divided into turret-type mounting machines and arch-type mounting machines, as shown in Table 2 [93] and Figure 10. The turret-type mounting machine has a fast mounting speed, but costs are high. The arch-type mounting machine has a slightly lower mounting speed but is suitable for a wide range of components and has good flexibility. Therefore, if the mounting sequence of the arch-type SMP machine is optimized, it can balance between low cost and high efficiency. The mounting cycle of the arch-type SMP machine mainly includes $D_{take}$, $D_{mount}$, and $D_{next}$, where $D_{take}$ represents the length of the pick-up path in a cycle, $D_{mount}$ represents the length of the patch path in a cycle, and $D_{next}$ represents the distance from the last mounting position of the current cycle to the initial position of the next cycle.

In the early stage, the related research only focused on the optimization of $D_{mount}$, that is, the optimization of the PCB placement sequence was regarded as a common TSP problem and the number of nuzzles was only one. Khoo et al. [94] developed a patch planning system based on the genetic algorithm, which used the OX crossover operator, reverse mutation operator, and exchange mutation operator to improve the genetic algorithm. Preliminary analysis showed that the moving distance was shortened by 19.80%. Subsequently, the researchers began considering the feeder’s position and the components’ mounting sequence and considered the replacement of nozzles, collision interference, workload (for multi-arm arch-type mounting machines), and other issues. Lee et al. [93] proposed a hierarchical method to improve the production efficiency of multi-head placement machines. This method divided the sequence optimization problem into three levels: construction of feeder-groups, assignment of feeder-groups, and sequencing of pick-and-place movements. Among them, the first two problems were solved by the heuristic algorithm based on dynamic programming and the third problem was solved by the heuristic algorithm based on the nearest neighbor method. The results showed that this method saved 18% time on average. Deo et al. [95] solved the complex problem of component sequence optimization in a multiple setup PCB assembly machine arrangement by using an improved genetic algorithm and solved the coding problem by dividing the chromosome fragments into two segments: the first segment represented the distribution of components on the feeder and the second segment represented the mounting sequence of components. This method achieved satisfactory results. Zhu et al. [96] proposed a meme triangular probability distribution shuffled frog-leaping algorithm, which overcame the defect of the traditional frog-leaping algorithm with fewer individual updates and gave the implementation forms of subtraction, addition, and multiplication for discrete problems. Under the premise that the position of the components on the feeder was determined, it achieved a higher convergence rate and higher accuracy than the hybrid leapfrog algorithm. Lin et al. [97] summarized the five significant issues faced by task planning for dual-arm placement machines and established a mathematical model. They balanced the load by reasonably partitioning PCBs, determined the number of various types of nozzles on the ANC (automatic nozzle changer) using the quantity ratio method, and proposed an improved differential evolution algorithm and a random-key encoding mapping method to optimize the feeder position. The experimental results showed that the assembly time of PCB was reduced by 13% at the most. In addition, some new intelligent algorithms have been applied to placement sequence optimization and satisfactory results have been obtained, such as the immune algorithm [98], the wolf pack algorithm [99], the cellular bat algorithm [100], the Hopfield neural network method [101], and so on.

#### 3.1.3. AOI Path Planning

When using SMT equipment to process PCB, it is necessary to carry out AOI before the product is completed to ensure the quality of the product. General quality problems include the wrong direction of components, a lack of welding of some components, and so on, significantly impacting electronic equipment’s performance stability. Before the emergence of AOI technology, manual visual inspection was generally used, which was inefficient and significantly affected by personal factors. At the beginning of the last century, some countries in Europe and the United States began to seek ways to replace visual inspection, which accelerated the development and maturity of AOI technology and made using AOI on SMT production lines an industry development trend [102]. The main factors affecting the AOI system’s efficiency are: (1) camera image acquisition and transmission speed, (2) speed of moving parts, and (3) image processing and pattern recognition speed [103]. Nowadays, with the development and progress of machine vision technology, the processing speed of factors 1 and 3 has been dramatically improved. The speed of moving parts limits the detection efficiency of the AOI system. Because the speed of the moving parts cannot be improved indefinitely and many factors restrict the camera’s FOV (field of view), optimizing the detection path becomes the key to solving the problem. In a typical AOI system, the motion mechanism drives the detection camera to complete the detection of components. Because the AOI detection is non-contact, the FOV is larger than the size of a single component to detect multiple components simultaneously. However, due to the restriction between the maximum FOV and the size of the PCB, it is impossible to complete the quality inspection of the whole PCB by taking only one or a few photos, which brings some difficulties to path planning.

**Figure 11:**
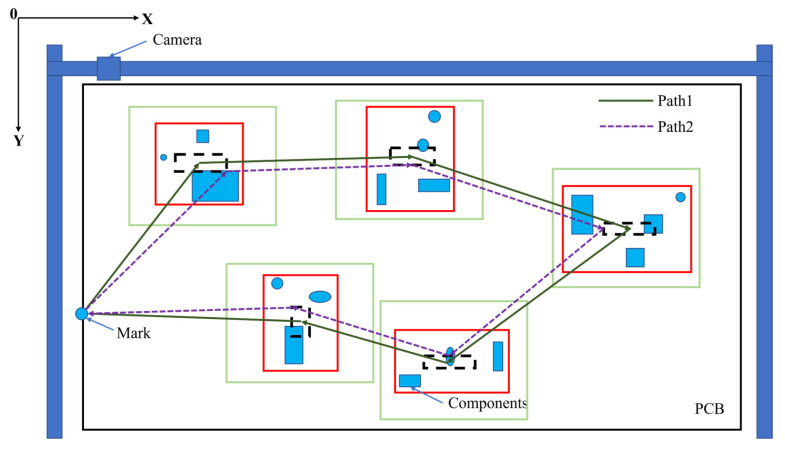
Adjustment domain of detection windows.

The AOI detection path optimization problem can be divided into component clustering and path optimization problems. Among them, component clustering needs to cover all components, with as few camera shots as possible, and path planning needs to minimize the path within the adjustable range of the center of the FOV. In the early days, researchers’ attention was limited to one of these, which led to an incomplete solution to the problem. Kim et al. [104] thought that the typical clustering algorithm could not be directly applied to the clustering of components in AOI detection; the problem lay in the limited size and variable number of clustering windows in AOI detection. Therefore, a clustering algorithm combined with genetic algorithm was proposed, which redefined the chromosome, fitness function, and crossover operator of the genetic algorithm. The experimental results showed that the clustering performance was improved by 4%. Huang et al. [105] proposed a new multi-directional search particle swarm optimization algorithm, in which the update of particle position took into account not only the personal best and the global best but also the information contained in other particles, which improved the search accuracy and obtained the minimum number of detection windows. Then, the researchers realized that the clustering problem and the path optimization problem were not two separate problems but coupled with each other. The small number of detection windows did not mean that the detection time was short, hence they needed to be considered together. Park et al. [106] established a total objective function for the AOI detection path problem using the hybrid genetic algorithm to determine the window location and detection path at the same time; the designed crossover, mutation, and sorting operators not only ensured the diversity of search but also contributed to fast convergence. The algorithm reduced the amount of clustering and the distance of detection movement and then improved the performance by 6~10%. Ma et al. [107] adopted a similar method to improve detection efficiency by up to 12%. The above techniques considered the combination of the detection window and components. Still, they did not consider adjusting the windows’ position because it had a specific flexible space; so, adjusting the window position would also change the detection efficiency. As shown in Figure 11, the solid green line represents the FOV range, the solid red line represents the component clustering result, and the black dotted line represents the adjustable range of the center of FOV. It can be seen from the figure that the path still has an extensive range of variation within the adjustment range. Zhong et al. [108,109,110] proposed a three-stage path-planning algorithm. In the first two stages of the algorithm, the incremental clustering algorithm and the genetic algorithm were used to form a preliminary detection path and the center position of the window was dynamically adjusted in the third stage. Thus, the number of windows and path length was reduced. Experiments showed that the algorithm was fast and easy to implement. Deng et al. [111] proposed an AOI path planning method based on the variable neighborhood ant colony algorithm. Aiming at the limitation of the ant colony algorithm, a variable neighborhood path search method with three kinds of neighborhood structure was proposed. A variable neighborhood window adjustment method was proposed to address the problem of image window position adjustment. The experimental results showed that the algorithm had higher efficiency and quality. Given the limitation that the ant colony algorithm could easily fall into the local optimal solution, Xiao et al. [112] proposed an improved ant colony algorithm based on a negative feedback pheromone update strategy, which reduced the average path length by 1.7%. Three location adjustment algorithms were proposed, which combined the improved ant colony algorithm with the location adjustment algorithm to shorten the path length by up to 13.7%.

PCBs are the core components of a variety of electronic equipment, especially in aerospace and radar electronic equipment. Because of its variety, small batch production and processing mode, efficient path planning algorithm, and reasonable path planning, results are significant. No matter the path planning problem of drilling, mounting, or AOI detection, it is essentially a TSP planning problem. With the expansion of the problem scale, the time cost will inevitably rise. Therefore, future research should also focus on new intelligent algorithms, improve and combine existing algorithms to some extent, or even apply deep learning and reinforcement learning to this problem, striving to achieve the best balance between accuracy and efficiency.

### 3.2. Assembly Trajectory Planning

**Figure 12:**
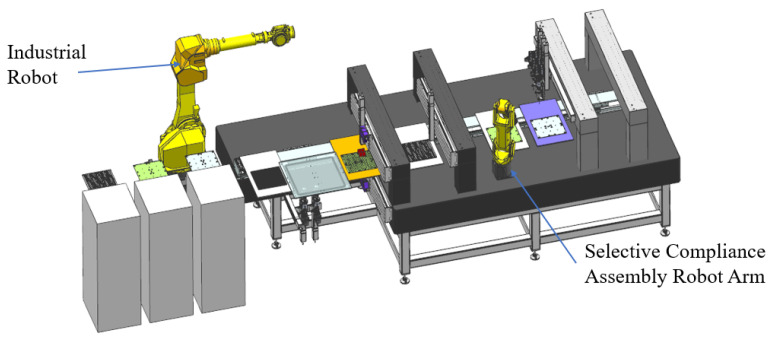
An array antenna assembly equipment.

**Figure 13:**
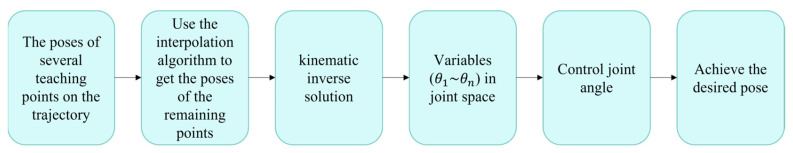
The routine process of trajectory planning.

Assembly is a crucial step in the production process of electronic equipment, which directly determines the quality and efficiency of the product. The industrial robot has been widely used in electronic equipment manufacturing and assembly because of its flexibility, good interaction ability, small space requirements, and 7 × 24-h working mode [113]. Take the array antenna assembly equipment shown in Figure 12 as an example, which needs to assemble the antenna board, the PCB, and the heat dissipation board. In the feeding stage and the screwing stage, industrial robots are used instead of workers, which improves work efficiency and reduces the possibility of artificial assembly errors. However, planning their trajectories is necessary to maximize the industrial robots’ efficiency. Industrial robot trajectory planning generally refers to the relationship between space coordinates and time at the robot’s end in joint or Cartesian space [114]. On the one hand, it can improve industrial robots’ processing efficiency and working accuracy and improve electronic equipment’s stability and service time. The reasonable trajectory can also reduce impact and energy consumption to a certain extent and prolong the service life of industrial robots [115]. The general flow of trajectory planning is shown in Figure 13.

Since there are countless options for the trajectory of the end of industrial robots from the starting point to the endpoint, trajectory planning can be divided into general trajectory planning and optimal trajectory planning, according to the purpose of trajectory planning [116]. General trajectory planning focuses more on the continuity of motion, which can be divided into joint space planning and Cartesian space planning. Optimal trajectory planning focuses on the optimization calculation of various targets, which can be divided into time, energy, and impact optimization. In this section, general trajectory planning and optimal trajectory planning are discussed, respectively, and relevant technologies are summarized at the end.

General trajectory planning uses various interpolation algorithms to plan the trajectory. Generally, it uses linear and circular interpolation as the basic interpolation methods to fit more complex motion trajectories. The processes for complex motion trajectories are polynomial fitting, B-spline fitting, NURBS curve fitting, etc. Polynomial fitting uses cubic, quintic, or seventh-degree polynomials to interpolate the curve planning. Fang et al. [117], based on kinematic analysis, adopted cubic polynomials and seventh-degree polynomials, respectively, to study the trajectory planning of robots. The results showed that the seventh-degree polynomials effectively solved the problem of discontinuous acceleration and obtained continuous smooth trajectory curves of each joint. Generally speaking, the higher the degree of a polynomial, the better the interpolation effect, but the Runge phenomenon is easy to appear. That is, large vibration is easy to appear at both ends of the interpolation region and the computational complexity is high [118]. B-spline fitting calculates the control points through the points determined in the known space to obtain the interpolation curve, which has the advantages of second-order continuity and symmetry, among which the cubic B-spline curve is the most commonly used [119]. Kong et al. [120] proposed an improved quintic polynomial interpolation method to solve the mutation problem caused by higher-order polynomials, which fused quintic polynomial interpolation with B-spline interpolation to eliminate the mutation phenomenon. The experiment showed that the optimized joint curve was smoother. However, the cubic B-spline curve did not solve the problem of large impact and discontinuity and was only applicable when interpolation points were closely spaced [121]. Given the limitations of cubic B-spline interpolation in terms of high dynamic performance, Guan et al. [122] proposed a method combining third-order and fourth-order polynomials to interpolate trajectories. The first and last segments of trajectories were interpolated with cubic polynomials and other parts were interpolated with cubic polynomials, which achieved a good application effect. In addition, NURBS curves could also be used to improve the trajectory. Yue et al. [123] studied the trajectory planning of industrial robots based on NURBS curves. They used NURBS to fit free curves and S-shaped velocity curves to control the velocity and acceleration at the robot’s end. In recent years, some researchers have begun to combine intelligent algorithms with fitting methods and some research results have been achieved. Han et al. [124] combined a piecewise polynomial interpolation algorithm with PSO. Firstly, piecewise polynomial interpolation was used to fit the trajectory. Then, PSO was used to optimize the trajectory, with time as a fitness function, which improved the working efficiency and ensured the stability of the whole operation.

**Figure 14:**
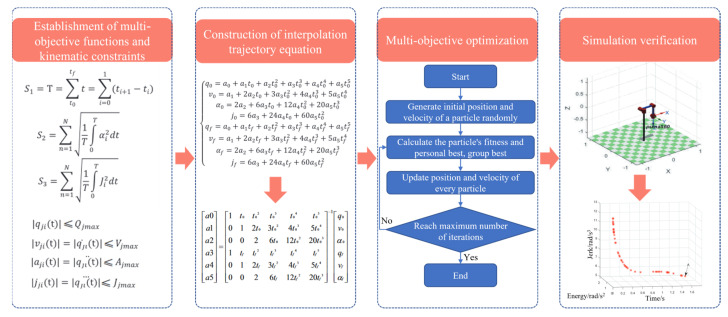
Optimal trajectory planning process [133].

Optimal trajectory planning believes that, in practical applications, it should not only consider trajectory but also pay more attention to such factors as energy consumption, stationarity, and work efficiency during robot operation. Therefore, optimal trajectory optimization methods targeting time, energy, and impact are generated [125,126,127]. Previously, researchers focused more on single-objective optimization methods. Zhang et al. [128] proposed a time-optimal and smooth trajectory planning algorithm, which utilized the time-optimal theory based on the dynamics model to plan the trajectory of the robot, construct the trajectory optimization model under the constraints of the geometric path and joint torque, and dynamically select the optimal trajectory parameters during the solving process to improve the robot’s motion speed significantly. The reliability of the algorithm was verified on a 6-DOF industrial robot. Li et al. [129] proposed an energy characteristic parameter model based on the dynamic time-scaling, aiming at the shortcoming of the traditional dynamic time programming method that required intensive computation. The energy consumption in the state transition process was described as a function of scaling parameters. Experiments showed that this method reduced energy consumption by 33.7%. Subsequently, researchers began to introduce various intelligent algorithms into the optimal trajectory planning to save computing time and improve computing speed. He et al. [130] proposed a time-optimal trajectory planning algorithm for robots based on the genetic algorithm. A seventh-degree B-spline curve was used to complete the interpolation of joint position-time series. The kinematic constraints were transformed into the constraints of a B-spline control vertex. The algorithm reduced the running time of the robot by 22%. Li et al. [131] used an improved sparrow algorithm to solve the problem of optimal energy consumption for robots. They also used a seventh-degree B-spline curve to construct a joint space trajectory and combined kinematics parameters and dynamics parameters to calculate the total energy consumption of the robot. They used various methods to improve the sparrow algorithm and solve the time series corresponding to the optimal energy consumption, thereby planning the trajectory of the optimal energy consumption and verifying its rationality through experiments. Since the single-objective optimization method only considers a single problem, it is often necessary to consider the multi-objective optimization problem in practical applications, i.e., hybrid optimal trajectory planning. Therefore, optimal trajectory planning for multiple objectives has become a hot research topic. Li et al. [132] proposed a hybrid algorithm (referred to as CSNSGA-II) based on the cuckoo search (CS) algorithm and the non-dominated sorting genetic algorithm-II (NSGA-II), which adopted a quintic non-uniform rational B-spline curve for trajectory planning and established a multi-objective optimization model with movement time, impact, and energy consumption as optimization objectives. Under the constraints of speed and acceleration, a hybrid algorithm was used to solve the problem and experiments verified its effectiveness. He et al. [133] proposed a multi-objective trajectory optimization method based on particle swarm optimization. The mathematical model of the working trajectory was established with a quintic polynomial and corresponding kinematic constraints were added, as shown in Figure 14. Other hybrid optimal trajectory planning algorithms also have similar processes, but the difference lies in the intelligent algorithm, the interpolation algorithm, and the optimization objective.

**Figure 15:**
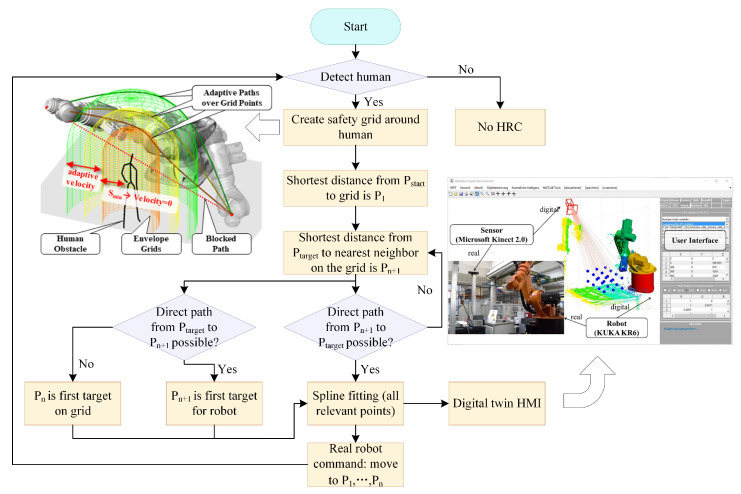
Reactive path planning algorithm [139].

Collision-free trajectory planning is a critical technology in Industry 4.0 and human-robot collaboration (HRC). Therefore, we should consider the static and dynamic obstacles while planning the path. Pellegrinelli et al. [134] studied the energy consumption of pick-and-place robots during product assembly. The probabilistic roadmap technique and the open robot realistic library (ORL) provided by COMAU were used to generate a collision-free trajectory based on obtaining the energy-optimized trajectory. Hu et al. [135] designed an improved Gaussian mixture model (GMM)/Gaussian mixture regression (GMR) -based approach for collaborative robots’ path planning. They used a dynamic recursive ant colony algorithm to speed up the iterative speed of GMM/GMR. The experiment proved that the method was robust and efficient. Zhao et al. [136] proposed a dynamic recursive ant colony algorithm and used the algorithm to solve the collision-free trajectory planning problem. They verified the practicability and validity of the method through simulation. To better avoid collisions, some researchers began to use AR technology for collision detection. Chen et al. [137] proposed a method for detecting collisions between a robot and a physical entity by comparing the depth values of the corresponding pixels in the depth image and the computer-generated image. This method could effectively obtain the collision-free path of the virtual robot. In the future, industrial robots are expected to share the same workspace with human workers. Therefore, in the process of human–robot collaboration, human–robot collision should also be avoided to protect both humans and robots from injury or damage. Safeea et al. [138] simplified human co-workers and robots into capsules and combined the concept of hypothetical repulsion and attraction vectors and a mathematical representation of robot’s kinematics to constantly correct the offline path during the movement so that the robot could avoid collision with human co-workers. Dröder et al. [139] built a digital twin platform using machine learning to innovatively surround humans in a virtual safety fence. The envelope grid would be dynamically adjusted according to the position of the person, thus forcing the robot to maintain a minimum safe distance. The safety fence, digital twin platform, and path planning algorithm are shown in Figure 15. Kanazawa et al. [140] extended the task scheduling system by installing an online trajectory generation system. Collision-free and time-shortest trajectory was calculated simultaneously based on the probabilistic prediction of the worker’s motion and the receding horizon scheme for the trajectory planning. The system could also deal with irregular behavior of workers. The above classification was based on the type of obstacles, which was more in line with the actual application in the industrial field. In addition, it could also be divided into classical approaches, graph-based and grid-based search approaches, and heuristic and meta-heuristic approaches according to the collision avoidance method [141].

Assembly is a crucial step in the manufacturing process of electronic equipment. The large-scale application of industrial robots raises the flexibility and efficiency of assembly to a new level [142]. Before assembly, it is usually necessary to carry out trajectory planning for the robot to obtain a working path conforming to the actual conditions [143]. This section elaborates on general trajectory planning and optimal trajectory planning of industrial robots. Among them, general trajectory planning is mainly carried out in joint spaces and has developed into a mature planning mode. In the future, interpolation methods such as high-order polynomials and spline curves will be used more to reduce impact. Optimal trajectory planning considers optimal planning under multiple objectives. With the emergence of more new optimization objectives in practical applications, optimal trajectory planning needs to be further improved. Additionally, trajectory collision checking is required to keep people and resources safe. In the future, trajectory planning will not only consider issues such as motion continuity, impact, and energy consumption, but will also need to further consider collision detection.

## 4. Force–Position Coordination Control

With the acceleration of competition in the electronic equipment industry, electronic equipment presents a development trend of high density, miniaturization, and integration, which brings great difficulties to the assembly of complex parts with diverse materials and irregular shapes. When performing the automatic assembly of small components, the shaft-hole assembly between the parts is mainly carried out. Various reasons will lead to assembly failure, including sensor measurement errors and manufacturing and installation errors of robots or machine tools, etc. Due to the complex coordination relationship between parts, tiny position errors or uncertainty may produce large contact force, resulting in deformation or damage to parts. To meet electronic equipment manufacturers’ increasingly stringent manufacturing tolerance requirements, the force control accuracy must ≤ ±0.1 N and the visual positioning error must ≤±30 μm. It is challenging to ensure assembly accuracy only through position error calibration and compensation, so the contact force needs to be controlled with high precision in the assembly process between shaft and hole. Some scholars have realized the flexible control of contact force in the intelligent assembly of electronic equipment from the perspective of mechanical structure design, for example, remote center compliance devices (RCC), etc. [144], but this method has low control accuracy and is easily interfered with by the external environment. At the same time, problems such as a lack of universality and poor portability of the compliant mechanism cannot be adapted to processing multiple types of workpieces [145].

As one of the critical technologies in the automated assembly process, the force–position coordination control strategy does not require the design of specialized compliant mechanisms. Instead, the actuator moves to the designated position by integrating force and position feedback information, achieving perfect coordination between electronic equipment components and increasing flexibility and adaptability in the automatic assembly process; therefore, it plays a decisive role in the intelligent assembly technology of electronic equipment. Currently, the force–position coordinated control methods used for the intelligent assembly of electronic equipment mainly include hybrid force–position control, impedance control, intelligent control, visual servo, etc. This section will detail the research status of several control strategies in the intelligent assembly of electronic equipment and the compliant assembly of micro and small components.

### 4.1. Hybrid Force–Position Control

**Figure 16:**
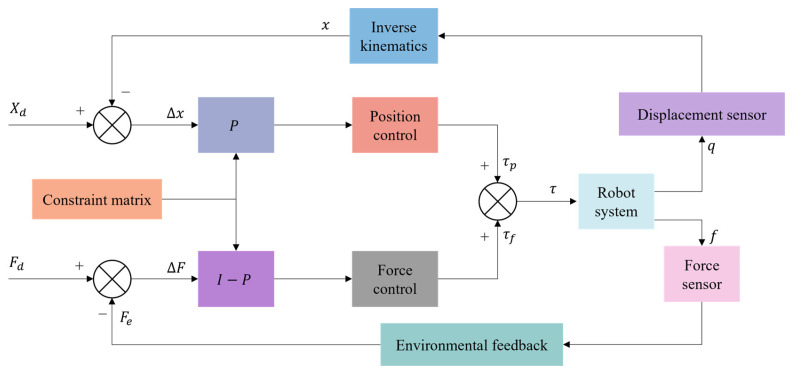
Hybrid force–position control.

Hybrid force–position control is to plan the processing task space into two mutually orthogonal subspaces of force control and position control and to control the two simultaneously through control strategies in an independent form. The control principle is shown in Figure 16. Hybrid force–position control was first proposed by Mason [146] in 1979, who believed that the robot could carry out force control and position control simultaneously. Based on Mason’s theory, Craig and Raibert [147] formally proposed the hybrid force–position control. They realized the force–position control of the manipulator in any direction by introducing the Jacobian matrix. However, this method required a large amount of calculation and was difficult to be applied in practice. Zhang et al. [148] simplified the calculation amount of the controller and proposed replacing the space position control loop of the robot end-effector with the joint position control loop.

In recent years, to meet the requirements of compliant assembly in the field of electronic equipment, the hybrid force–position control has been gradually applied to the axle hole assembly of small-size parts. Hu et al. [149] proposed a robot assembly scheme combining vision and force perception to meet the assembly requirements of special satellite parts. They adopted the hybrid force–position control method to carry out soft steering control on the pin, realizing the workpiece’s accurate assembly and meeting the project’s implementation requirements. Yang et al. [150] proposed a new hybrid force–position control strategy to solve the problem of assembly failure caused by different inclination angles between axes and holes in precision assembly and improved the selection matrix to reliably complete the assembly task. Park et al. [151] successfully realized the axis-hole assembly with a gap of 50 μm by combining the hybrid force–position control with passive compliance control. Rakotondrabe et al. [152] carry out the hybrid force–position control of a pick-and-release task using the microgripper to prove the interest of the developed microgripper and the proposed control scheme.

Hybrid force–position control is one of the most common control strategies. However, due to the problems of slow control response speed, environmental modeling, and determining control strategies, the current hybrid force–position control is still being continuously improved and revised in its control theory. The practical application in the field of intelligent assembly of electronic equipment has yet to be further developed.

### 4.2. Impedance Control

**Figure 17:**
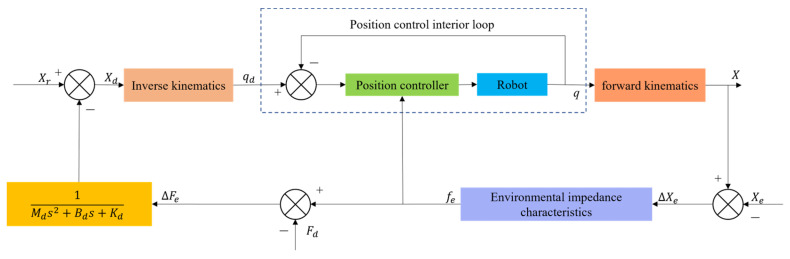
Position-based impedance control.

**Figure 18:**
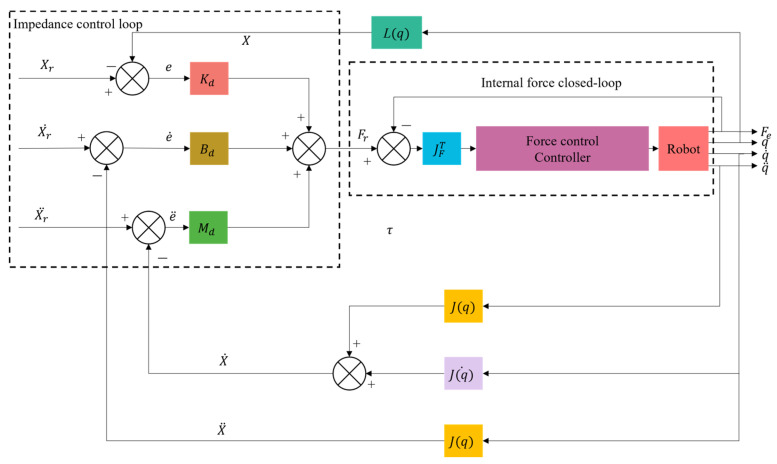
Torque-based impedance control.

Impedance control has been improved based on hybrid force–position control, generally divided into position-based impedance control (Figure 17) and torque-based impedance control (Figure 18). Unlike hybrid force–position control, impedance control realizes the unity of free and constrained environments. Force control can be realized indirectly by adjusting impedance control parameters. It was first proposed by Whitney et al. [153,154] to adjust the position of the end-effector of the mechanism by establishing a dynamic model between the contact force of the end-effector and the deviation of the position to achieve the compliant control of the end. Based on the previous work, Hogan et al. [155] made a comprehensive summary and induction of impedance control. They proposed an impedance control method based on mechanism dynamics, which successfully solved the jamming problem in shaft-hole assembly and promoted the development of impedance control.

In precision operations such as PCB board assembly, T/R component (used in phased array antennas) assembly, and electrical connector insertion, the impedance control strategy has been applied. Krüger et al. [156] applied impedance control in robot shaft-hole assembly to actively adjust the position and pose according to the contact force between axes and holes. Song et al. [157] proposed an admittance control strategy based on visual force information for assembling parts of small sizes and arbitrary irregular shapes. Appropriate admittance controller parameters were selected through force/torque feedback to compensate for positioning errors. Mol et al. [158], of the European Space Agency, proposed a closed-loop force sensor-based nested admittance/impedance control strategy. The stability bounds on the control parameters of this method were established through numerical simulation and the task of shaft-hole assembly of millimeter parts was realized. Chen et al. [159], from the University of Texas, proposed a robust impedance control algorithm. They applied it to the assembly task of inserting a PCB into a connector socket and proved its feasibility. Zhang et al. [160] analyzed the biaxial assembly’s contact state and contact force, proposed fuzzy force control strategies, and realized the millimeter-level hole assembly task. Shuang et al. [161] suggested a DDPG (deep deterministic policy gradient) -based variable parameter admittance control algorithm integrated with fuzzy reward mechanism to complete the robot peg-in-hole assembly task in an unstructured environment and conducted experiments on five holes with different diameters to achieve flexible peg-in-hole assembly. Song et al. [162] proposed a method for the electric connector-mating process in a wiring harness assembly system, which combined visual servo and impedance control to achieve compliant control in the assembly process. Cho et al. [163] proposed an electrical connector assembly strategy by an industrial robot to realize impedance control based on a six-axis force/torque sensor.

### 4.3. Intelligent Control

**Figure 19:**
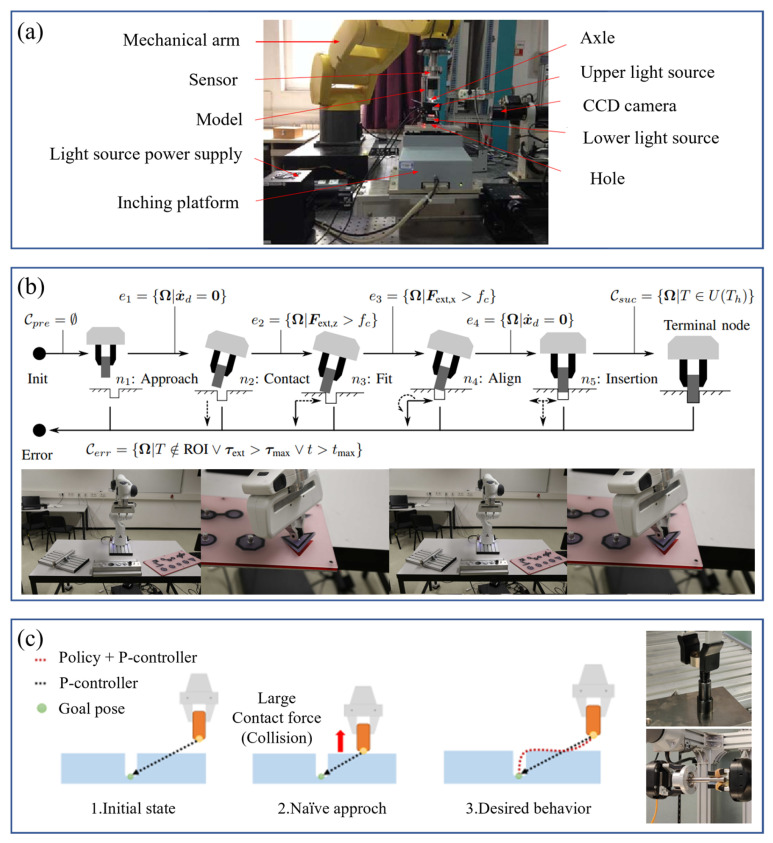
Research and application of intelligent control: (**a**) shaft-hole assembly based on BP natural network and genetic algorithm; (**b**) adaptive impedance control assembly based on deep reinforcement learning; (**c**) variable admittance parameter shaft-hole assembly based on reinforcement learning [167,169,170].

In recent years, the traditional single-control strategy has been increasingly challenging to meet the demand. Whether it is a hybrid force–position control, an impedance control, or an adaptive control for the intense coupling characteristics of force control, modeling analysis of the contact environment is required. All possible assembly states are considered in advance. Generally, these methods can achieve good control effects only on the premise of good identification ability to the environment. However, with the complexity of assembly types, these methods are often unable to adapt to the unstructured environment of intelligent assembly of electronic equipment. Compared with the traditional control strategy, intelligent control can use a smart control algorithm to process the data information fed back by sensors, enabling the robot to realize self-regulation and adaptation in the case of unknown assembly environmental conditions and improve the execution results after continuous self-learning to complete the assembly task. With the advancement of artificial intelligence algorithms, the intelligent control strategy has been widely used in automatic assembly. At present, the mainstream intelligent control strategies mainly include neural network control (including BP (back propagation) neural network, RBF (radial basis function) neural network, recursive neural network, etc.), fuzzy logic control, intelligent algorithms (including the particle swarm optimization algorithm, the genetic algorithm, the ant colony algorithm, the simulated annealing algorithm, etc.). Many scholars have conducted in-depth research on this issue. Jakovljevic et al. [164] proposed a fuzzy inference mechanism for identifying the state of the assembly between the axis and hole and realized the prediction of the contact state. Fan et al. [165] proposed an adaptive fuzzy genetic learning algorithm to update controller parameters and control actuator position and contact force, thus improving control accuracy and robustness of the control system. Babaci et al. [166] adopted fuzzy control rules to adjust the changing relationship between the reference and command positions in impedance control. The task of peg-in-hole assembly was realized by adjusting the position of the robot’s end to improve the assembly accuracy. Li [167] researched the assembly of small and precise parts. In the contact stage, the BP neural network and the genetic algorithm were used to establish a micro-force feedback model to realize the shaft-hole assembly with 2 μm and 5 μm assembly gaps, as shown in Figure 19a.

Due to its powerful learning and adaptability, deep reinforcement learning has gradually become a research hotspot in the field of high-precision automatic assembly. Liu et al. [168] proposed a self-learning method based on deep reinforcement learning to solve the problem that traditional robots relied on a large amount of manual programming and complex coding. Deep neural networks were used to encode robot control strategies and realize shaft-hole assembly under environmental uncertainties. Johannsmeier et al. [169] learned the meta parameters in the adaptive impedance controller through deep reinforcement learning technology. As shown in Figure 19b, they realized the submillimeter axis-hole assembly task. Beltran-Hernandez et al. [170] used the reinforcement learning method to learn time-varying parameters of admittance control from the experiential data of robot interaction with the environment. They realized the task of axis-hole assembly with a gap of 0.05 mm. The control principle and results are shown in Figure 19c. Inoue et al. [171] adopted the deep reinforcement learning algorithm to complete the screw insertion task of a narrow gap under the limitation of robot positioning accuracy and sensor accuracy. Fan et al. [172] combined deep reinforcement learning with supervised learning to realize the task of high-precision shaft-hole assembly. Ren et al. [173] established a deep reinforcement learning model for high-precision shaft-hole assembly and realized the task of pin-hole precision assembly. Wang et al. [174] proposed a dynamic assembly algorithm based on deep reinforcement learning against the complex and dynamic noise disturbances in the dynamic assembly environment and verified it in real scenes. Luo et al. [175] incorporated the force/torque information of the operating space into reinforcement learning, obtained the precise control parameters of the variable impedance force, and completed the high-precision assembly of gear sets. Wu et al. [176] addressed the problem of traditional precision assembly methods that relied on robot system programming by using deep reinforcement learning networks for prioritization to achieve learning of nail-in-hole insertion skills.

### 4.4. Visual Servo

As early as the 1970s, machine vision was first proposed and, in the following decades, machine vision technology developed rapidly. Machine vision’s control mode was divided into “look-and-move” and visual servo control modes. The former converts the target position obtained by the vision system into the robot coordinate system. The robot moves to the designated place to complete the assembly, essentially belonging to the open-loop control mode. The visual servo mode is to obtain the target image through visual feedback, compare it with the given target image, and adjust the posture of the end-effector with the image deviation to realize the assembly task. To put it simply, the vision system is used as a servo mechanism to realize real-time control of the position error. This section mainly discusses the visual servo control mode.

**Figure 20:**
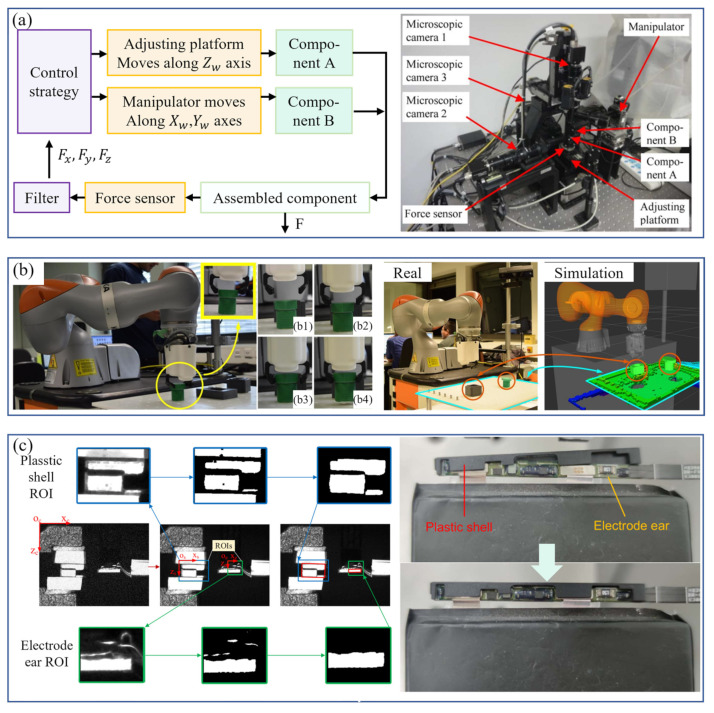
Research status of visual servo control: (**a**) high-precision shaft-hole assembly based on micro-vision and force information; (**b**) shaft-hole assembly with 3D and 2D visual information, (**b1**–**b4**) represent grasping of object based on precise position computed from the vision system (**c**) position–force visual servo control based on monocular vision [178,179,184].

From the perspective of the sensing mode, impedance control, hybrid force–position control, and intelligent control all belong to assembly controlled by forces. In contrast, visual servo control belongs to compliance control guided by vision. Its detection precision is high and the technology is relatively mature. Meanwhile, visual servo technology can be combined with intelligent decision making or machine-learning algorithms, which have been widely used to assemble small parts. Chen et al. [177] studied the compliant assembly strategy combining 3D vision and force perception to solve the problem of compliant assembly of parts at any position and posture in assembly. They realized the assembly of parts with a fitting gap of 0.5 mm. Liu et al. [178] developed a high-precision automatic assembly system based on microscopic vision and force information to complete the task of axis-hole assembly in the size of millimeter level with an interference fit (Figure 20a). Le et al. [179] established a robot assembly system integrating 3D and 2D visual information. They realized the assembly task of peg-in-hole assembly with a diameter of about 10 mm (Figure 20b). Chang et al. [180] developed a visual servo micro-assembly control method based on dynamic position through image calibration, regional scanning with edge-fitting, and shadow-aided positioning algorithms. Aiming at the precise assembly problem, Liu et al. [181] realized the robot shaft-hole assembly task based on the visual servo. Kim et al. [182] used computer vision to assemble parts with unknown cross-sectional shapes, estimated and compensated for assembly errors, and conducted insertion experiments on circular and square holes. Zhang [183] designed a set of precision assembly systems based on binocular vision measurement to realize the assembly process of tiny axle-hole assembly without contact. Chen et al. [184] proposed a position–force visual sensing method based on a single-lens imaging system in an eye-to-hand configuration because of some problems in assembling thin-walled deformable objects in robotic peg-in-hole assembly. Experiments showed that the nonlinearity of the visual force sensing was about 3% in the whole range and the assembly effect was good (Figure 20c). Sun et al. [185] developed a robot wiring harness assembly system based on vision and force/torque sensors to realize the mating of electric connectors. The feasibility of this scheme was proven through experiments. Wang et al. [186] proposed an intelligent recognition and assembly guidance method for aerospace electrical connectors based on vision. Combining offline image training and online part area recognition, three-dimensional part information and assembly path information were integrated into an assembly information guidance model. Finally, assembly personnel were guided to complete the assembly of electrical connectors. The test results showed that the recognition accuracy and assembly efficiency significantly improved.

The above research shows that visual servo control has brought tremendous potential to the automatic assembly of small parts in electronic equipment and has shown significant advantages in improving the accuracy and efficiency of parts assembly. However, the current visual servo technology still has some limitations. Firstly, the sensor equipment, such as the camera, needs to be calibrated in advance and the position to be processed in the process of taking photos requires no occlusion in the field of vision. In addition, deep information is hard to obtain. Further, due to the non-contact measurement, the visual servo control cannot detect the force generated by contact between parts, which is highly likely to cause damage to equipment or workpieces in case of sudden collision or interference. Considering the development trend of miniaturization and the miniaturization of terminal products in electronic equipment, visual servo technology can only be applied to some non-contact scenes in the intelligent assembly of electronic equipment.

The above force–position coordination control methods are summarized in Table 3.

Flexible, precise, and high-quality assembly of complex parts is a challenging problem in electronic equipment assembly. The large-scale application of intelligent assembly can greatly benefit the field of electronic equipment, which has better consistency than a large number of manual assemblies. However, the unbalanced development of assembly precision, component strength, hardware precision, and algorithm robustness has seriously hindered the development of force–position coordinated control technology. This section analyzes complex parts’ assembly, small parts’ assembly, and shaft-hole assembly in the intelligent assembly of electronic equipment and summarizes the principle and application of force–position coordination control. This section also analyzes the advantages and disadvantages of different control strategies and their application scenarios.

## 5. Conclusions

As the assembly of electronic equipment shifts from manual assembly to intelligent and automated assembly, visual positioning, path and trajectory planning, and force–position coordination control will all play an important role. This article summarizes the application of the above three critical technologies in the intelligent assembly of electronic equipment in detail. In the future, the development of these technologies will mainly include the following aspects:

Machine vision is increasingly used in intelligent assembly production lines due to its advantages, such as low equipment cost, simple structure, and good real-time performance. In the future, electronic assembly technology will develop in the direction of high processing accuracy and assembly efficiency. Therefore, the research and improvement of positioning devices and algorithms must also develop to achieve accuracy and efficiency. The main points are: (1) Accurate recognition in complex scenes. The assembly components of electronic equipment are becoming increasingly intelligent and complex, forming complex and dynamic processing procedures. The recognition rate and accuracy of visual recognition technology may be slightly reduced, so optimizing the recognition and positioning algorithms in complex scenes is necessary. (2) Achieving miniaturization, high stability, and high integration. When designing the intelligent assembly of electronic equipment, it is essential to consider the overall dimensions, FOV, calibration, and other visual system issues. A miniaturized, highly stable, and highly integrated visual system can better match robots, SMP machines, AOI detection systems, and other equipment for assembly work, optimizing the workflow, simplifying the equipment structure, and improving assembly efficiency.

Robot trajectory planning is one of the critical research issues in the robotics industry today. In the face of increasingly complex assembly requirements, it is developing towards high accuracy, high efficiency, low energy consumption, and low impact. The future development directions are as follows: (1) The current trajectory planning mainly faces offline scenarios and the effect verification is conducted chiefly using software for simulation; however, in reality, due to various errors, the actual trajectory often differs from the theoretical trajectory. Therefore, it is possible to use visual or external sensors to obtain accurate postures and conduct real-time trajectory planning. (2) As the complexity of industrial robot application scenarios gradually increases, more and more factors need to be considered in practical applications. How to integrate new optimization goals and add them to hybrid optimal planning is also a matter of concern. For PCB processing path optimization, the following aspects can be started in the future: (1) continue to seek new swarm intelligence algorithms to solve various problems existing in current intelligent algorithms; (2) seek a combination of multiple intelligent algorithms to overcome the shortcomings of a single intelligent algorithm with composite algorithms; (3) with the help of multi-core processors, new designs and implementations of existing algorithms are implemented to solve problems in a multithreaded manner, improving the speed and accuracy of the solution.

Assembly is a critical step in electronic equipment manufacturing and force–position coordination control plays an important role. The future research directions of intelligent assembly force–position coordination control technology for electronic equipment include: (1) Combining traditional control strategies with intelligent control algorithms. The combination of the two is relatively simple and intelligent control is only used as compensation for conventional control strategies. In the future, we can consider combining the two more reasonably. (2) Multi-source sensor data fusion technology. Currently, compliance control strategies mainly rely on a single sense of force or visual information feedback and can only be applied to simple specific scenarios. In the future, multi-source sensing information such as vision and force perception can be integrated to improve the perception ability of actuators to unstructured environments to enhance their robustness and stability.

As one of the core technologies of Industry 4.0, intelligent assembly technology has practical applications in various scenarios, such as automobile body assembly, engine assembly, and assembly of electronic equipment, as described in this paper. With the development of intelligent assembly technology, its application scope will gradually expand. In addition to the future development of the three key technical points mentioned above, intelligent assembly technology should also pay attention to the following aspects: (1) With the development of Industry 4.0, the close collaboration between humans and robots may bring additional risks. It is necessary to expand the application field of human–machine collaboration to ensure safety, so that the robot becomes an active partner rather than a passive mechanical servant. (2) Constant changes in markets, products, and regulations, as well as the emergence of mass customization, require intelligent assembly systems to achieve greater flexibility and adaptability. This can expand the scale of production, increase the variety of products, and better meet the demand. (3) Virtual assembly technology, including augmented reality (AR) and digital twin (DT), realizes the verification and optimization of the assembly process in a virtual environment. The application of virtual assembly technology in intelligent assembly can significantly improve production efficiency and reduce production and operation costs. (4) A smart factory is an intelligent, automated, and digital manufacturing system and all equipment will be networked and smart. In such a highly automated scenario, attention must be paid to network reliability, device autonomy, and various potential security issues.

## Figures and Tables

**Table 1:** Summary of common machine vision measuring equipment.

Equipment	Advantages	Limitations
Monocular vision	Simple structure, high integration, small size, low cost, a small amount of calculation	Capable of measuring 2D information, challenging to obtain the depth and pose information
Binocular vision	Accurate positioning, high-quality dynamic shooting, capable of calculating 3D position coordinates	High error matching rate in a complex environment, high requirements in the data transmission capability
Multi-vision	Capable of establishing large-scale visual measurement fields, more accurate and more intelligent	Complicated calibration, lots of measurement data, difficulty in data processing

**Table 2:** Classification of the SMP machine [93].

Type		Number of Nozzles	Speed	Price	Operating Difficulty
Turret-type		1~16	High	High	Simple
Arch-type	Single head	1~3	Medium	Low	Medium
	Double heads	(1~3) × 2	High	Medium	Difficult
	Multiple heads	4~32	High	Low	Very Difficult

**Table 3:** Comparison of force–position coordination control methods.

Control Strategy	Advantages	Limitations	Applicable Scenarios
Hybrid force–position control	High precision of force control	Slow response speed, the environment needs to be modeled accurately	Precision assembly and occasions where components have complex structures, uncertain positions, and are prone to generate large contact forces
Impedance control	Capable of inhibiting vibration, a small amount of computation, high stability and flexibility	Necessary to establish an accurate model of control object, poor adaptability	
Intelligent control	Capable of learning hidden control strategies from date, a wide range of applications, strong real-time performance	Long training time, potential environmental interference issues	
Visual servo	Low cost, low noise, excellent safety, robustness, and stability	Easily affected by illumination, occlusion, camera accuracy	Large workpiece gaps and small contact forces, low accuracy requirements

## Data Availability

Not applicable.
